# Impact of propofol versus sevoflurane on the incidence of postoperative delirium in elderly patients after spine surgery: study protocol of a randomized controlled trial

**DOI:** 10.1186/s13063-022-06687-x

**Published:** 2022-08-30

**Authors:** Ji-hua Wang, Meng Lv, Hai-xia Zhang, Yang Gao, Ting-ting Chen, Tian-tian Wan, Yue-lan Wang

**Affiliations:** 1 Department of Anesthesiology, The First Affiliated Hospital of Shandong First Medical University & Shandong Provincial Qianfoshan Hospital, Ji’nan, 250000 Shandong China; 2 Shandong First Medical University, Ji’nan, 250000 Shandong China

**Keywords:** Delirium, Propofol, Sevoflurane, Spine surgery, Randomized controlled trial

## Abstract

**Background:**

Postoperative delirium in elderly patients is a common and costly complication after surgery. Propofol and sevoflurane are commonly used anesthetics during general anesthesia, and the sedative and anti-inflammatory mechanisms of the two medications are different. The aim of this trial is to compare the impact of propofol with sevoflurane on the incidence of postoperative delirium in elderly patients after spine surgery.

**Methods:**

A single-center randomized controlled trial will be performed at First Affiliated Hospital of Shandong First Medical University, China. A total of 298 participants will be enrolled in the study and randomized to propofol infusion or sevoflurane inhalation groups. The primary outcome is the incidence of delirium within 7 days after surgery. Secondary outcomes include the day of postoperative delirium onset, duration (time from first to last delirium-positive day), and total delirium-positive days among patients who developed delirium; tracheal intubation time in PACU; the length of stay in PACU; the rate of postoperative shivering; the rate of postoperative nausea and vomiting; the rate of emergence agitation; pain severity; QoR40 at the first day after surgery; the length of stay in hospital after surgery; and the incidence of non-delirium complications within 30 days after surgery.

**Discussion:**

The primary objective of this study is to compare the impact of propofol and sevoflurane on the incidence of postoperative delirium for elderly patients undergoing spine surgery. The results may help inform strategies to the optimal selection of maintenance drugs for general anesthesia in elderly patients undergoing spine surgery.

**Trial registration:**

ClinicalTrials.govNCT05158998. Registered on 14 December 2021

## Administrative information

Note: the numbers in curly brackets in this protocol refer to SPIRIT checklist item numbers. The order of the items has been modified to group similar items (see http://www.equator-network.org/reporting-guidelines/spirit-2013-statement-defining-standard-protocol-items-for-clinical-trials/).

**Table:** 

Title {1}	Impact of propofol versus sevoflurane on the incidence of postoperative delirium in elderly patients after spine surgery: study protocol of a randomized controlled trial
Trial registration {2a and 2b}.	NCT05158998 [ClinicalTrials.gov]; 14 December 2021
Protocol version {3}	Version 1.1 of 08-30-2021
Funding {4}	This work was funded by the National Natural Science Foundation of China (82070078), and Academic Promotion Programme of Shandong First Medical University (2019QL015). The Foundation does not have any specific rights related to the publication of the results. No researchers involved in this trial have an economic interest in the Foundation or other financial supporters.
Author details {5a}	Ji-hua Wang Department of Anesthesiology, The First Affiliated Hospital of Shandong First Medical University & Shandong Provincial Qianfoshan Hospital, Ji'nan, Shandong 250000, China Meng Lv Department of Anesthesiology, The First Affiliated Hospital of Shandong First Medical University & Shandong Provincial Qianfoshan Hospital, Ji'nan, Shandong 250000, China Hai-xia Zhang Department of Anesthesiology, The First Affiliated Hospital of Shandong First Medical University & Shandong Provincial Qianfoshan Hospital, Ji'nan, Shandong 250000, China Yang Gao Department of Anesthesiology, The First Affiliated Hospital of Shandong First Medical University & Shandong Provincial Qianfoshan Hospital, Ji'nan, Shandong 250000, China; Shandong First Medical University, Ji'nan, Shandong 250000, China Ting-ting Chen Department of Anesthesiology, The First Affiliated Hospital of Shandong First Medical University & Shandong Provincial Qianfoshan Hospital, Ji'nan, Shandong 250000, China; Shandong First Medical University, Ji'nan, Shandong 250000, China Tian-tian Wan Department of Anesthesiology, The First Affiliated Hospital of Shandong First Medical University & Shandong Provincial Qianfoshan Hospital, Ji'nan, Shandong 250000, China Yue-lan Wang Department of Anesthesiology, The First Affiliated Hospital of Shandong First Medical University & Shandong Provincial Qianfoshan Hospital, Ji'nan, Shandong 250000, China
Name and contact information for the trial sponsor {5b}	Investigator initiated clinical trial; Yue-lan Wang (Principal Investigator) wyldgf@163.com
Role of sponsor {5c}	This is an investigator initiated clinical trial. Therefore, the funders played no role in the design of the study and data collection, analysis, interpretation of data, and manuscript writing.

## Introduction

### Background and rationale {6a}

Postoperative delirium is one of the most common complications after surgery, especially in elderly individuals who now account for one-third of all surgical patients [1]. Postoperative delirium, which is characterized by acute onset of impaired cognitive function and inability to focus or sustain attention, delayed return to functional baseline, resulting in increased hospital length of stay and costs [2, 3]. Patients with postoperative delirium have an increased risk of cognitive and functional decline, incident dementia, and a 2–20-fold increased risk of mortality [4, 5], with hospital mortality rates of 25–33% [6, 7]. A survey of surgical specialists carried out by the American Geriatrics Society Geriatrics-for-Specialists Initiative (AGS-GSI) identified delirium as the most “essential” topic in the care of older adults and as the least understood geriatric clinical issue [8].

Spine surgery is the third most common surgical procedure in the elderly population [9]. The prevalence rate of postoperative delirium in geriatric patients undergoing spine surgery is reported to be 12.5–24.3% [10–12]. One study of the development of postoperative delirium after spine surgery found that the average length of stay increased to over 7 days [13]. Despite its significance, there are few effective treatment strategies. Thus, prevention of delirium is essential [14].

The pathophysiological mechanisms of delirium remain poorly understood; neurotransmitter imbalance and neuroinflammation are the leading models. Precipitating factors include sedative-hypnotic agent, anesthesia, surgery, pain, anemia, infections, acute illness, and acute exacerbation of chronic illness [15]. Propofol and sevoflurane are commonly used anesthetics to maintain sedation during spine surgery, and induce unconsciousness through different mechanisms [16]. Previous studies have found that propofol and sevoflurane have different anti-inflammatory effects [17, 18]. Considering the different sedative and anti-inflammatory effects, propofol and sevoflurane may have different effects on postoperative delirium. There are many studies to compare the effects of propofol with sevoflurane on postoperative delirium. However, the conclusions are controversial [19–24]. Therefore, we designed a randomized, controlled, double-blind clinical study to compare the effect of propofol and sevoflurane on delirium after spine surgery in elderly patients.

## Objectives {7}

To compare the effect of propofol and sevoflurane on the rate of delirium after spine surgery in elderly patients

## Trial design {8}

This is a single-center, double-blind, exploratory, randomized controlled trial with two parallel arms. Eligible patients will be enrolled and randomly assigned to receive either sevoflurane-based inhalational anesthesia or propofol-based intravenous anesthesia.

## Methods: participants, interventions, and outcomes

### Study setting {9}

The study will be conducted in the First Affiliated Hospital of Shandong First Medical University, China.

### Eligibility criteria {10}

#### Inclusion criteria

Participants will be included if they meet all of the following criteria: (1) age ≥ 65 years and ≤ 90 years; (2) scheduled to undergo spine surgery, under general anesthesia; (3) American Society of Anesthesiology (ASA) I–III; and (4) give signed written informed consents.

#### Exclusion criteria

(1) Family history or history of malignant hyperthermia; (2) history of propofol or sevoflurane allergy; (3) demonstrated cognitive impairment on the modified Mini-Mental State Examination (MMSE) (score, <24 of 30 or < 20 of 30 if the patient’s education year was less than 6 years or<17 if the patient is illiterate); (4) planned postponed tracheal extubation or transferred to ICU; (5) severe visual or auditory handicap; (6) prior diagnoses of neurologic diseases or mental disorders (e.g., stroke, Parkinson’s disease, dementia, schizophrenia, or depressive illness); (7) take anticholinergic drugs or other drugs acting on the central nervous system for a long time before operation; and (8) participating in other clinical studies within 3 months.

### Who will take informed consent? {26a}

The day before surgery, investigators who have been trained for the study procedure will screen potential participants according to the inclusion and exclusion criteria, explain the contents of informed consent in detail to the patients who met the criteria and their families, and obtain the informed consent of the patients or their authorized clients.

### Additional consent provisions for collection and use of participant data and biological specimens {26b}

Not applicable, no additional consent will be obtained as this trial does not involve collecting biological specimens for storage.

## Interventions

### Explanation for the choice of comparators {6b}

Propofol and sevoflurane are commonly used anesthetics, and the different effects of the two drugs on postoperative delirium are controversial. Thus, we want to compare the effect of the two drugs on the incidence of delirium.

### Intervention description {11a}

#### Anesthesia management and study intervention

Intraoperative monitoring includes an electrocardiogram (ECG), invasive blood pressure (IBP), pulse oxygen saturation (SPO_2_), bispectral index (BIS), end-tidal partial pressure of carbon dioxide (PetCO_2_), end-tidal concentration of inhalational anesthetics, cerebral oxygen saturation, and urine output.

Participants will be induced with midazolam (0.03–0.06mg/kg), etomidate (0.05–0.3mg/kg), sufentanil (0.2–0.5μg/kg), and atracurium (0.5mg/kg) intravenously. For patients in the sevoflurane group, anesthesia will be maintained with sevoflurane inhalation, of which the concentration will be adjusted to maintain the BIS value between 40 and 60. For patients in the propofol group, anesthesia will be maintained with propofol infusion (target-controlled infusion, TCI), of which the target concentration will be adjusted to maintain the BIS value between 40 and 60. For patients of both groups, intraoperative analgesia will be maintained with remifentanil (0.1–0.5μg kg min^−1^) and muscle relaxation will be maintained with continuous infusion of atracurium (10 μg kg min^−1^).

Intraoperative mechanical ventilation will be established with a tidal volume between 6 and 8 mL/kg, plateau pressure of <40 cm H_2_O, frequency between 12 and 16 per min, and FIO_2_: 60%. The PetCO_2_ will be maintained between 35 and 45 mmHg. Intraoperative fluid therapy will be managed according to routine practice. A blood cell saver will be used if it is estimated that the bleeding exceeds 500 mL during the operation. Packed red blood cells will be transfused if necessary in order to maintain hemoglobin levels within the target range of 7–10 g/dL. Vasoactive drugs (dopamine, norepinephrine, phenylephrine, urapidil, or nitroglycerin) will be administered if necessary in order to maintain the systolic blood pressure fluctuation range within 30% of baseline. After the operation, the patients will be transferred to the postanesthesia care unit (PACU).

When suturing the skin, sufentanil (10μg) will be given to analgesia and the atracurium will be stopped. Postoperative analgesia during the first 2 days will be provided by a patient-controlled analgesia pump, which is established with sufentanil (1.5–2 μg/mL) and ondansetron (0.16 mg/mL), programmed to deliver a 0.5-mL bolus with a lockout interval of 15min and a background infusion of 2mL/h. Other opiates and non-steroid anti-inflammatory drugs can also be used for postoperative analgesia. Postoperative rescue analgesia will be provided by intravenous injection or oral intake.

Intraoperative data included the type of surgery (categorized the complexity and invasiveness of the surgical procedure according to an established four-tier rating system [25]); durations of anesthesia and surgery; dosages of anesthetics, analgesics, and other drugs used during anesthesia; fluid balance and transfusion of blood products; and fluctuation of monitoring variables.

### Criteria for discontinuing or modifying allocated interventions {11b}

Patients can refuse to continue the study at any time for any reason without any consequences. The patient’s participation in this study can also be ended by the investigator if the patient is allergic to propofol or sevoflurane during surgery, is uncooperative, or cannot be assessed (e.g., endotracheal intubation or coma) with a 3-min diagnostic interview for Confusion Assessment Method (3D-CAM) after surgery. The patient data that have been collected up to that moment will be included in the analysis.

### Strategies to improve adherence to interventions {11c}

Not applicable. Interventions in this study will be completed during general anesthesia and without the patients’ cooperation.

### Relevant concomitant care permitted or prohibited during the trial {11d}

No pre-anesthesia medication will be given before the patient arrived in the operation room. For all enrolled patients, scopolamine and penehyclidine are prohibited; atropine is used only for the purpose of reversing bradycardia, and the dosage will be recorded.

### Provisions for post-trial care {30}

Not applicable. In the present study, the intervention measures administered for patients of both groups are anesthesia methods currently being used during daily practice. Patients will be managed according to routine practice.

### Outcomes {12}

#### Primary outcome

The incidence of postoperative delirium in two groups. Delirium will be assessed twice each day, that is, between 8–10 am and 6–8 pm, during postoperative days 1–7 with 3D-CAM. For patients who are discharged from the hospital, withdraw consents, or die within 7 days after surgery, the last follow-up results will be regarded as the final results. Because of the waxing and waning nature of delirium, researchers will review all progress notes and nursing documentation for delirium diagnoses, and a thorough medical record review process using the Chart-based Delirium Identification Instrument [26] will be performed.

#### Secondary outcome

If the patient develops postoperative delirium, the day of postoperative delirium onset, duration (time from first to last delirium-positive day), and total delirium-positive days among patients will be recorded. The tracheal intubation time, incidence of postoperative shivering, incidence of postoperative nausea and vomiting (PONV), incidence of emergence agitation (EA), and pain severity will be assessed during the recovery period in PACU. Postoperative shivering will be assessed with the Crossley and Mahajan Scale (0, no shivering; 1, one or more of the following: piloerection, peripheral vasoconstriction, and peripheralcyanosis with no other cause, but no muscle activity; 2, visible muscular activity confined to one muscle group; 3, visible muscular activity in more than one muscle; 4, gross muscular activity involving the whole body) [27]. Nausea is defined as a subjective, unpleasant sensation associated with awareness of the urge to vomit. Retching is defined as the labored, spastic, rhythmic contraction of the respiratory muscles without expulsion of the gastric contents. Vomiting is defined as the forceful expulsion of gastric contents from the mouth. Each episode is recorded as either presence or absence. If the patients had nausea, the severity will be recorded using the following scale: 1, mild nausea; 2, moderate nausea; and 3, severe nausea. If the patients had retching or vomiting, the severity of episodes will be recorded using the following scale: 1, one episode; 2, two episodes; and 3, three or more episodes [28]. PONV are also assessed during postoperative days 1–3. EA will be assessed immediately after extubation using the Richmond Agitation–Sedation Scale (RASS) and patients with RASS score > +1 are evaluated as EA [29]. RASS are also assessed in patients who developed delirium to define the types of delirium (patients with delirium are classified into three subtypes: hyperactive (RASS score consistently positive, from +1 to +4), hypoactive (RASS score consistently neutral or negative, from −3 to 0), and mixed) [30]. Pain severity will be assessed with the visual analogue scale (VAS), when the patients are transferred out of PACU and during postoperative days 1–3.

Postoperative recovery quality on the first day after operation will be assessed with QoR40. The occurrence of non-delirium complications, which are defined as newly occurring medical conditions that are harmful for patients’ recovery and require therapeutic intervention, will be monitored during postoperative days 1–30. For patients who die within 30 days after surgery, the exact date of death will be recorded.

### Participant timeline {13}

**Fig. 1:**
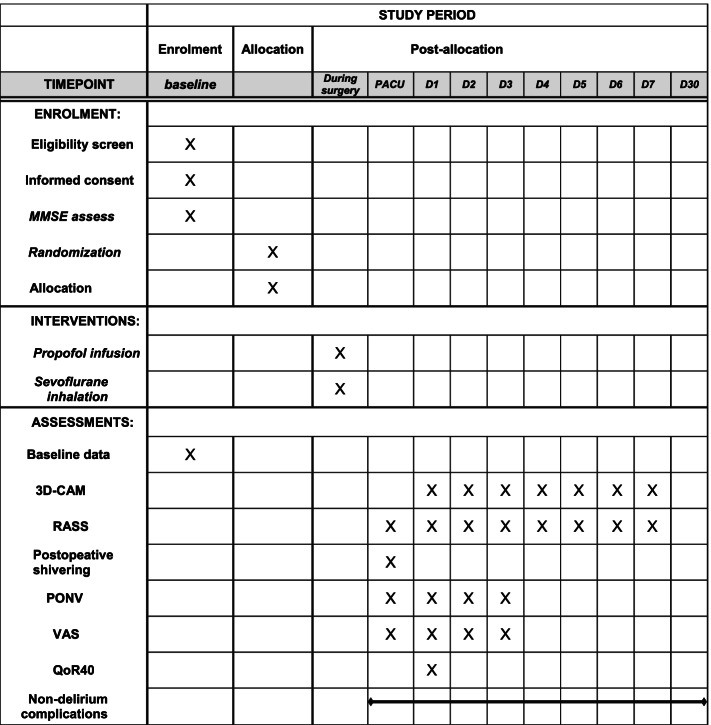
Participant timeline

The participant timeline is presented in Fig. 1.

### Sample size {14}

The sample size was calculated for the primary outcome (the incidence of delirium after spine surgery) based on a retrospective study investigating the risk factors of delirium after spine surgery [31]. Calculations are performed using PASS 15.0 and the parameters used included the following: a two-sided, significance level of 0.05, 95% confidence interval, 80% power, and 1:1 allocation. The minimum number of subjects required to confirm statistical significance is determined to be 134 individuals per group. Accounting for a dropout rate of 10%, we aim to recruit a total of 298 people, with 149 for each group.

### Recruitment {15}

Patients will be recruited at the First Affiliated Hospital of Shandong First Medical University, China. More than 400 elderly patients undergo spine surgery in the center annually.

## Assignment of interventions: allocation

### Sequence generation {16a}

SPSS software version 25.0 will be used to generate a random number table with a 1:1 ratio to one of the two groups by a statistician, who will not be involved in patient assessments.

### Concealment mechanism {16b}

To implement blindness, an opaque, sealed, sequentially numbered envelope will be assigned to each patient after randomization and will be opened by the anesthesia staff who is not involved in the study.

### Implementation {16c}

After obtaining written informed consent, the research nurse will assign the participants to one of the two groups according to the random numbers. An opaque, sealed, sequentially numbered envelope will be given to the anesthesia providers before surgery by the nurse.

## Assignment of interventions: blinding

### Who will be blinded {17a}

Anesthesia providers who are in charge of the intraoperative management of the participants will not be blinded. Participants, researchers, and follow-up personnel will be blinded.

### Procedure for unblinding if needed {17b}

Not applicable. Anesthesia providers are not blinded and deal with the adverse events of the anesthetics.

## Data collection and management

### Plans for assessment and collection of outcomes {18a}

Data will be collected from electronic patient records or by asking the patient and collected with a case report form (CRF). For patients who have been discharged, a telephone follow-up will be used to collect complications within 30 days after surgery.

For recruited patients, baseline data will be collected. These include demographic data (age, gender, education level, body mass index, living alone or not, history of smoking and drinking) and previous histories of diseases (comorbidity, medical treatment, surgical history, and history of postoperative delirium), the results of laboratory and instrumental examinations, the severity of comorbid disorders (assessed with Charlson comorbidity index), and the evaluation of physical status (assessed with the American Society of Anesthesiologists physical status classification).

Patients will be assessed with the scale (MMSE, RASS, 3D-CAM, VAS, Crossley and Mahajan Scale, QoR40) by the researcher.

To guarantee consistent assessment, researchers are uniformly trained.

### Plans to promote participant retention and complete follow-up {18b}

During the preoperative visit, researchers will explain the procedure of intraoperative management and postoperative follow-up in detail. The researchers will let the patients think about whether they can complete the follow-up and then decide whether to participate in the study. To promote participant retention and complete follow-up, the addresses and telephone numbers of participants and their relatives are collected after obtaining consents.

### Data management {19}

The CRF and assess scales will be anonymized and saved in a safe. Only the study team has access to this safe. Data will be entered into an electronic database. Only the study team has access to the electronical data. Different data entering individuals will use standardized terminology and abbreviations, and training will be performed regarding entering data on forms. Any missing data or errors in the data will be summarized along with detailed descriptions and will be queried by checking the original forms. To guarantee a uniform assessment, researchers are uniformly trained.

### Confidentiality {27}

Research data will be stored using a study identification code for each participant. The key to the identification code list will only be available to the research team during the study and will be documented and safeguarded by the principal investigator according to research guidelines after completion of the study. No patient identification details will be reported in publications.

### Plans for collection, laboratory evaluation, and storage of biological specimens for genetic or molecular analysis in this trial/future use {33}

Not applicable. There are no biological specimens in this study.

## Statistical methods

### Statistical methods for primary and secondary outcomes {20a}

Data will be analyzed by using SPSS (25.0). Data from the primary outcomes (the incidence of delirium) will be presented as categorical variables. To compare between the two treatment groups, a *χ*^2^ test with an intention-to-treat analysis will be performed. Secondary outcomes in patients with delirium compared delirium characteristics using Fisher exact testing between the two groups. Continuous data will be presented (mean±standard deviation (SD)) and compared between the two groups using *t* tests if the data is a normal distribution. Continuous data will be summarized (median and interquartile range [IQR]) and compared between the two groups using no-parametric tests if the data is not a normal distribution. Categorical data will be summarized as frequencies (percentages) and compared between study groups using *χ*^2^ tests or Fisher exact tests, as appropriate. A multivariable generalized linear regression analysis models the effect of baseline variables on the incidence relative risk of delirium.

### Interim analyses {21b}

Not applicable. There are no interim analyses planned.

### Methods for additional analyses (e.g., subgroup analyses) {20b}

Not applicable. There are no subgroup analyses planned.

### Methods in analysis to handle protocol non-adherence and any statistical methods to handle missing data {20c}

All randomized participants will be included in the main analyses. An intention-to-treat analysis will be carried out relating to any protocol non-adherence or dropouts. Per-protocol dataset will also be performed for any of the missing data. Besides, multiple imputation will be performed to handle missing data.

### Plans to give access to the full protocol, participant-level data, and statistical code {31c}

This study protocol is publicly available on www.clinicaltrials.gov. The datasets analyzed during the current study and statistical code are available from the corresponding author on reasonable request, as is the full protocol.

## Oversight and monitoring

### Composition of the coordinating center and trial steering committee {5d}

This is a single-center study, performed and coordinated in the First Affiliated Hospital of Shandong First Medical University, China. Day-to-day support for the trial is provided by:

- The principal investigator: takes supervision of the trial and medical responsibility of the patients.
- The data manager: organizes data capture and safeguards quality and data.
- The study coordinator: trial registration, coordinates study visits and annual safety reports.
- The participant evaluator: identifies potential recruits, takes informed consent, and collects the baseline data.
- The study nurse: randomization.
- The study assessor: the follow-up of the patients and collects the data after surgery.

The study team meets weekly. There is no trial steering committee or stakeholder and public involvement group

### Composition of the data monitoring committee, its role, and reporting structure {21a}

Not applicable. There are no data monitoring committees in this study. This is a low-risk study and the data is collected by researchers who are independent from the sponsor. All the researchers in the study declare that they have no competing interests.

### Adverse event reporting and harms {22}

In this study, the interventions in both groups are routine anesthesia methods during daily clinical work. Therefore, our study will not cause additional risk for the participants. However, even routine anesthesia may result in adverse events. In such cases, anesthesiologists will manage patients according to routine practice. The occurrence of adverse events will be monitored from the beginning of anesthesia to 24 h after surgery. Any adverse events will be treated immediately according to routine practice, and the patients will be followed up until it is completely resolved or therapy is terminated. Severe adverse events will be reported to the local ethical committee as soon as possible.

### Frequency and plans for auditing trial conduct {23}

The department of research office of the First Affiliated Hospital of Shandong First Medical University and the ethics committee of the First Affiliated Hospital of Shandong First Medical University will be able to audit the study at their own discretion (at least once a year). They would check the presence and completeness of the investigation file, which include informed consents, inclusion and exclusion criteria, source data, protocol violation and adverse events, research progress, and other files.

### Plans for communicating important protocol amendments to relevant parties (e.g., trial participants, ethical committees) {25}

Any protocol amendments will be written into a formal substantial amendment and will be approved by the ethics committee before implementation.

## Dissemination plans {31a}

The results of this study will be published completely in international peer-reviewed journals. Both positive and negative results will be reported.

## Discussion

This is a prospective, single-center, randomized, parallel-group, double-blind, controlled trial aimed to compare the impact of propofol and sevoflurane on the incidence of postoperative delirium for elderly patients undergoing spine surgery. The implementation of randomization and allocation concealment is well designed in this study. The results may help optimize the selection strategy of maintenance drugs for general anesthesia in elderly patients undergoing spinal surgery.

Our study also has several limitations. First, delirium will be just assessed twice each day, which may lead to some patients who developed delirium and recovered between the twice assessment omitted. Although a thorough medical record review process using the Chart-based Delirium Identification Instrument will be used to assess delirium between the twice assessment, different assessments may lead to heterogeneity, and sensitivity analysis will be used. Second, this is a single-center randomized controlled trial; thus, the external validity is limited.

## Trial status

Patient recruitment will start in January 2022. The current protocol is version 1.1 of 30-08-2021. Patient recruitment is estimated to be completed around November 2024.

## Abbreviations

ASA: American Society of Anesthesiology
BIS: Bispectral index
3D-CAM: 3-min diagnostic interview for Confusion Assessment Method
ECG: Electrocardiogram
EA: Emergence agitation
IBP: Invasive blood pressure
PONV: Postoperative nausea and vomiting
PetCO_2_: End-tidal partial pressure of carbon dioxide
PACU: Postanesthesia care unit
RASS: Richmond Agitation–Sedation Scale
SPO_2_: Pulse oxygenation saturation
VAS: Visual analogue scale
CRF: Case report form
MMSE: Mini-Mental State Examination
